# Prolonged hospitalization in Brazil: clinical need or systemic failure?

**DOI:** 10.1590/1806-9282.20250913

**Published:** 2025-10-27

**Authors:** Cassiano Teixeira

**Affiliations:** 1 Universidade Federal de Ciências da Saúde de Porto Alegre, Medical School, Department of Internal Medicine and Rehabilitation Sciences – Porto Alegre (RS), Brazil.

**Keywords:** Length of stay, Hospitalization, Health system, Delayed diagnosis, Health services accessibility

## Abstract

**BACKGROUND:**

Prolonged hospitalization increases the risk of adverse events and resource overuse. In dual public-private systems like Brazil's, it is unclear how much hospital length of stay reflects clinical needs versus systemic delays.

**OBJECTIVE:**

The aim of the study was to quantify clinical and non-clinical contributions to length of stay in medical inpatients and compare patterns between public (Unified Health System [SUS]) and privately insured patients.

**METHODS:**

We conducted a prospective multicenter cohort study including 5,423 adults admitted via the emergency department to internal medicine wards in three Brazilian hospitals (2009–2022). Physicians recorded daily whether ongoing hospitalization was clinically necessary or due to delays (e.g., tests, specialist input, administrative or social issues). Statistical analyses included multivariate models adjusted for comorbidities and frailty.

**RESULTS:**

The mean length of stay was 11.8±5.2 days, but only 38% (4.5±2.6 days) were clinically justified. The remainder resulted from non-clinical delays, particularly in diagnostics (2.9±3.1 days) and specialist input (1.9±1.5 days). SUS patients had longer stays than those privately insured (13.2 vs. 10.1 days; p<0.001), despite similar clinical complexity. These differences remained significant after adjustment. SUS patients were more likely to stay ≥12 days (OR 1.76), wait ≥3 days for tests (OR 1.89), and ≥2 days for specialist evaluations (OR 1.67).

**CONCLUSION:**

Most hospital days were not due to clinical needs. Diagnostic and specialist delays, especially in the public system, were key contributors to prolonged length of stay, highlighting structural inefficiencies and the need for system-wide reforms.

## INTRODUCTION

Hospital length of stay (LOS) is widely used as an indicator of hospital efficiency, care quality, and patient outcomes. Yet, prolonged hospitalizations are strongly associated with adverse effects such as nosocomial infections, medication-related events, physical deconditioning, and institutionalization—especially in older or frail individuals^1,2^. These risks increase incrementally with each additional day, highlighting LOS as a key issue in both safety and resource allocation^3,4^.

Although patients with greater clinical complexity are expected to stay longer, recent evidence suggests that logistical issues—like delays in diagnostics, specialist consultations, administrative clearance, and discharge coordination—may have an equal or greater impact on LOS^5,6^. Brazil's dual health system, composed of the public Unified Health System (SUS) and private insurers, presents a unique setting to assess how systemic factors shape hospital trajectories. While SUS serves most of the population, challenges such as limited resources and centralized workflows may slow care delivery. In contrast, private systems tend to provide quicker access to diagnostic and specialist services. However, few studies have compared the weight of clinical versus non-clinical drivers of LOS within this structure, particularly among medical inpatients.

This study addresses that gap by tracking over 5,000 patients in three hospitals over 4 years, recording daily reasons for continued hospitalization. We hypothesized that public system patients would be more exposed to systemic delays, leading to longer stays not explained solely by medical conditions.

## METHODS

### Study design and setting

This was a multicenter, prospective observational cohort study conducted between January 1, 2009, and December 31, 2022, in three general hospitals located in southern Brazil. All participating institutions were medium-to-large tertiary centers with active emergency departments and internal medicine wards. Each hospital included in the study had an established electronic medical records (EMR) system, multidisciplinary clinical teams, and standardized daily clinical rounds led by board-certified internists.

The study was approved by the ethics committees of all participating hospitals and followed national and international research guidelines involving human subjects. As only de-identified data were used for analysis, the requirement for informed consent was waived.

### Participants

We included all adult patients (≥18 years old) who were admitted via the emergency department to internal medicine teams. Only index hospitalizations were considered; readmissions during the study period were excluded to avoid duplicate outcomes. Patients admitted for elective procedures, transferred directly to subspecialty or surgical teams, or managed by consulting services rather than primary internal medicine teams were excluded. Admissions were stratified according to healthcare funding source: public system (SUS) or private insurance.

Baseline patient information, comorbidity profiles, functional and frailty status, and previous healthcare utilization (e.g., recent hospitalizations or long-term care residency) were recorded at the time of admission.

### Data collection and variables

Data collection was performed prospectively using a standardized case report form (CRF) embedded into each hospital's EMR system. Attending physicians and trained residents documented key patient-level data during daily rounds, which were subsequently audited by research coordinators.

Each inpatient day was assigned to one of five mutually exclusive delay categories based on structured team discussion and clinical documentation: (a) Clinical necessity—This category refers to situations where patients present signs or symptoms of clinical instability, such as altered vital signs, worsening of their general condition, or complications associated with their primary illness. Such instability may necessitate continuous monitoring, therapy adjustments, or additional interventions to stabilize the patient. These cases often involve the administration of specific drugs (e.g., antibiotics, chemotherapy) or decisions regarding further diagnostic tests or specific procedures. (b) Waiting for diagnostic tests—Describes patients who have not yet undergone all necessary tests or are awaiting results for more than 24 h. These may include imaging studies (e.g., X-rays, computed tomography [CT] scans, magnetic resonance imaging [MRIs]) or laboratory tests (e.g., blood work, urinalysis, biopsies). Delays might arise due to service availability, processing time, or the complexity of test analyses. (c) Waiting for specialist consultation—Indicates that patients have been waiting for over 24 h for a consultation with a specific medical specialist, such as a cardiologist, neurologist, general surgeon, or urologist. Such evaluations are often pivotal for diagnosis, treatment adjustments, or planning future interventions. The delay may be linked to the specialist's schedule, the complexity of the case, or the need for multiple interdisciplinary assessments. (d) Administrative or insurance clearance delays—Refers to cases where patients or hospitals are awaiting approval for over 24 h for specific medical materials or procedures. These include medical devices, prosthetics, specific medications, or other resources essential for patient care. Delays may stem from bureaucratic processes, financial authorizations, or stock availability. (e) Delays due to social service processes—Relates to patients ready for discharge but reliant on social services to arrange essential equipment and services, causing delays of more than 24 h. Examples include home ventilation devices, hospital beds, oxygen concentrators, or other supportive care items. Social services liaise with suppliers, family members, and other stakeholders to ensure patients receive adequate care post-discharge.

Each delay was logged in days (rounded to one decimal), and cumulative time spent in each category was calculated per patient.

### Outcomes

The primary outcome was total hospital LOS, defined as the number of calendar days from admission to discharge or in-hospital death.

Secondary outcomes included in-hospital mortality, days attributable to each delay category, occurrence of complications [delirium, pressure injuries, falls, dialysis-requiring acute kidney injury (AKI), intensive care unit (ICU) transfer after 24 h], and receipt of exclusive palliative care during admission.

In addition, prolonged hospitalization was defined a priori as LOS ≥12 days (above the 75th percentile of the cohort). Similarly, relevant logistical delays were defined as ≥3 days waiting for diagnostics and ≥2 days waiting for consultation.

### Statistical analysis

All analyses were conducted using R (v4.3.0) and SPSS (v26.0, IBM Corp.). Continuous variables were reported as means (standard deviation [SD]) or medians (interquartile range [IQR]), based on distribution assessed via the Shapiro-Wilk test. Between-group comparisons used Student's t-test or the Mann-Whitney U test for non-normal data. Categorical variables were presented as counts and percentages, with comparisons via chi-square or Fisher's exact test.

To explore whether prolonged LOS was more related to clinical severity or systemic delays, we used a three-step modeling strategy. First, stratified analyses by tertiles of the Charlson Comorbidity Index and Clinical Frailty Scale (CFS) were performed, comparing LOS and delay patterns between SUS and private patients using two-way ANOVA with interaction terms. Second, multivariate linear regression assessed independent predictors of total LOS, using type of admission, age, sex, Charlson score, CFS, and cumulative delay days as covariates. Models were built sequentially: unadjusted (Model 1), adjusted for clinical factors (Model 2), and additionally for delays (Model 3). Multicollinearity was assessed (Variance Inflation Factor [VIF]<5). Finally, binary logistic regression estimated ORs for prolonged LOS (≥12 days), diagnostic delay (≥3 days), and consultation delay (≥2 days), adjusted for relevant covariates. Model calibration was checked using Hosmer-Lemeshow tests and area under the curve (AUC).

All p-values were two-tailed, with significance set at p<0.05. Missing data were minimal (<2%) and addressed by pairwise deletion.

## RESULTS

A total of 5,423 adult patients were included, with 2,432 (44.8%) admitted through private insurance and 2,991 (55.2%) through the public health system (SUS). Baseline characteristics were similar in age (61.8±21.4 vs. 61.2±18.5 years; p=0.276) and female sex distribution (44.4 vs. 43.8%; p=0.580). However, SUS patients had a slightly higher Charlson Comorbidity Index (3.4±2.1 vs. 3.2±2.4; p=0.004) and lower CFS scores (3.3±1.8 vs. 3.9±2.2; p<0.001). No significant differences were found in prior hospitalization (10.8 vs. 9.8%; p=0.220) or direct transfers from nursing homes (5.9 vs. 5.5%; p=0.470). The rate of in-hospital complications—such as delirium, pressure injuries, falls, dialysis-requiring AKI, and ICU transfer after 24 h—did not differ significantly between groups, although patients with private insurance underwent more surgical procedures (13.9 vs. 11.9%; p=0.030). In-hospital mortality was modestly higher among SUS patients (6.5 vs. 5.2%; p=0.040), and the rate of exclusive palliative care did not differ significantly (9.1 vs. 7.8%; p=0.070) (Table 1).

**Table 1:** Characteristics of medical patients admitted through the emergency department to internal medicine teams (n=5,423) based in type of hospital admission.

Characteristics		Type of admission		p-value
		Private insurance (n=2,432)	Public health system (n=2,991)	
Age, mean±SD		61.8±21.4	61.2±18.5	0.276
Female gender, n (%)		1,081 (44.4%)	1,310 (43.8%)	0.580
Charlson Comorbidity Index, mean±SD		3.2±2.4	3.4±2.1	0.004
Clinical Frailty Scale, mean±SD		3.9±2.2	3.3±1.8	**<0.001**
Hospitalization in the past 90 days, n (%)		238 (9.8%)	323 (10.8%)	0.220
Direct transfer from a nursing home, n (%)		134 (5.5%)	177 (5.9%)	0.470
In-hospital complications, n (%)				
	Delirium	290 (11.9%)	371 (12.4%)	0.540
	Pressure injuries	114 (4.7%)	171 (5.7%)	0.080
	Falls	144 (5.9%)	159 (5.3%)	0.320
	Acute kidney injury requiring dialysis	170 (7.0%)	242 (8.1%)	0.090
	ICU transfer after 24 h of hospital admission	309 (12.7%)	341 (11.4%)	0.180
	Surgical intervention required during hospitalization, n (%)	338 (13.9%)	356 (11.9%)	0.030
	Length of hospital stay (days), mean±SD	10.1±4.6	13.2±5.2	**<0.001**
	Exclusive palliative care defined during hospitalization, n (%)	190 (7.8%)	272 (9.1%)	0.070
	In-hospital mortality, n (%)	127 (5.2%)	194 (6.5)	**0.040**

Definitions and explanations: Charlson Comorbidity Index: This is a widely used tool for quantifying the burden of comorbidities in patients and predicting long-term mortality. The index assigns specific weights to 19 medical conditions based on their association with mortality. Clinical Frailty Scale: Designed by Rockwood et al., this tool evaluates frailty through clinical judgment, considering factors like mobility, energy, physical activity, and pre-hospital functioning. The scale generates a score ranging from 1 (very fit) to 9 (terminally ill). Complications during hospitalization: These complications were pre-defined before the study and included delirium, pressure injuries, falls, AKI requiring dialysis, and ICU transfer after the first 24 h of hospital admission. Notes: SD: standard deviation; ICU: intensive care unit. Indicates variability in the sample. Charlson Comorbidity Index and Frailty Scale: Provide insights into patient complexity and risk stratification. Statistically significant p-values are indicated in bold.

Across the entire cohort, most hospitalization days were attributable to non-clinical factors. While the mean LOS was 11.8±5.2 days, only 4.5±2.6 days were due to clinical necessity. In contrast, delays related to diagnostic tests (2.9±3.1 days), specialist consultations (1.9±1.5 days), administrative clearance (0.9±1.2 days), and social service processes (0.2±0.6 days) together accounted for 62% of the total hospital stay (Table 2).

**Table 2:** Distribution of hospitalization days.

Hospitalization days	Due to clinical reasons	Due to waiting for the diagnostic	Due to waiting for a Specialist consultation	Due to administrative or insurance	Due to social service processes
11.8±5.2	4.5±2.6	2.9±3.1	1.9±1.5	0.9±1.2	0.2±0.6

Due to clinical reasons: This category refers to situations where patients present signs or symptoms of clinical instability, such as altered vital signs, worsening of their general condition, or complications associated with their primary illness. Such instability may necessitate continuous monitoring, therapy adjustments, or additional interventions to stabilize the patient. These cases often involve the administration of specific drugs (e.g., antibiotics, chemotherapy) or decisions regarding further diagnostic tests or specific procedures. Due to waiting for the diagnostic: Describes patients who have not yet undergone all necessary tests or are awaiting results for more than 24 h. These may include imaging studies (e.g., X-rays, CT scans, MRIs) or laboratory tests (e.g., blood work, urinalysis, biopsies). Delays might arise due to service availability, processing time, or the complexity of test analyses. Due to waiting for a specialist consultation: Indicates that patients have been waiting for over 24 h for a consultation with a specific medical specialist, such as a cardiologist, neurologist, general surgeon, or urologist. Such evaluations are often pivotal for diagnosis, treatment adjustments, or planning future interventions. The delay may be linked to the specialist's schedule, the complexity of the case, or the need for multiple interdisciplinary assessments. Due to administrative or insurance: Refers to cases where patients or hospitals are awaiting approval for over 24 h for specific medical materials or procedures. These include medical devices, prosthetics, specific medications, or other resources essential for patient care. Delays may stem from bureaucratic processes, financial authorizations, or stock availability. Due to social service processes: Relates to patients ready for discharge but reliant on social services to arrange essential equipment and services, causing delays of more than 24 h. Examples include home ventilation devices, hospital beds, oxygen concentrators, or other supportive care items. Social services liaise with suppliers, family members, and other stakeholders to ensure patients receive adequate care post-discharge.

The mean length of hospital stay (LOS) for the entire cohort was 11.8±5.2 days. SUS patients had significantly longer hospitalizations compared to those with private insurance (13.2±5.2 vs. 10.1±4.6 days; p<0.001), despite a comparable number of days attributed to clinical reasons (4.6±2.6 vs. 4.3±2.5 days; p=0.074) (Table 3). The excess LOS in the SUS group was largely due to longer waits for diagnostic tests (3.5±3.0 vs. 2.1±2.8 days; p<0.001) and specialist consultations (2.3±1.5 vs. 1.5±1.4 days; p<0.001). Delays related to administrative clearance or social services were rare and did not differ significantly between groups.

**Table 3:** Hospital stay delay reasons by type of admission.

Delay reason	Mean±SD (days)		p-value
	Private insurance	Public health system	
Total hospitalization days	10.1±4.6	13.2±5.2	<0.001
Due to clinical reasons	4.3±2.5	4.6±2.6	0.074
Due to waiting for the diagnostic	2.1±2.8	3.5±3.0	<0.001
Due to waiting for a specialist consultation	1.5±1.4	2.3±1.5	<0.001
Due to administrative or insurance	0.8±1.2	0.9±1.1	0.220
Due to social service processes	0.1±0.5	0.2±0.6	0.112

Due to clinical reasons: This category refers to situations where patients present signs or symptoms of clinical instability, such as altered vital signs, worsening of their general condition, or complications associated with their primary illness. Such instability may necessitate continuous monitoring, therapy adjustments, or additional interventions to stabilize the patient. These cases often involve the administration of specific drugs (e.g., antibiotics, chemotherapy) or decisions regarding further diagnostic tests or specific procedures. Due to waiting for the diagnostic: Describes patients who have not yet undergone all necessary tests or are awaiting results for more than 24 h. These may include imaging studies (e.g., X-rays, CT scans, MRIs) or laboratory tests (e.g., blood work, urinalysis, biopsies). Delays might arise due to service availability, processing time, or the complexity of test analyses. Due to waiting for a specialist consultation: Indicates that patients have been waiting for over 24 h for a consultation with a specific medical specialist, such as a cardiologist, neurologist, general surgeon, or urologist. Such evaluations are often pivotal for diagnosis, treatment adjustments, or planning future interventions. The delay may be linked to the specialist's schedule, the complexity of the case, or the need for multiple interdisciplinary assessments. Due to administrative or insurance: Refers to cases where patients or hospitals are awaiting approval for over 24 h for specific medical materials or procedures. These include medical devices, prosthetics, specific medications, or other resources essential for patient care. Delays may stem from bureaucratic processes, financial authorizations, or stock availability. Due to social service processes: Relates to patients ready for discharge but reliant on social services to arrange essential equipment and services, causing delays of more than 24 h. Examples include home ventilation devices, hospital beds, oxygen concentrators, or other supportive care items. Social services liaise with suppliers, family members, and other stakeholders to ensure patients receive adequate care post-discharge. SD: standard deviation.

To better quantify these differences, we used clinically meaningful thresholds: ≥12 days for total LOS, ≥3 days waiting for diagnostic tests, and ≥2 days for specialist consultation. SUS patients had higher odds of prolonged hospitalization (OR 1.76), delays in diagnostic testing (OR 1.89), and delays in consultation (OR 1.67), compared to privately insured patients.

A sensitivity analysis was performed to determine whether these differences were due to baseline clinical status. Even after stratifying patients by frailty and comorbidity tertiles, SUS patients consistently had longer hospital stays. Among non-frail patients (CFS≤3), the gap remained pronounced (12.7±4.9 vs. 9.8±4.2 days; p<0.001). Multivariate linear regression models confirmed that public system admission was independently associated with prolonged LOS, even after adjusting for age, sex, comorbidity, frailty, and delay categories.

## DISCUSSION

This study showed that prolonged hospitalizations among Brazilian medical inpatients are largely driven by non-clinical delays rather than illness severity. Most hospital days were unrelated to active medical needs, regardless of insurance type. However, patients in the public health system (SUS) consistently stayed longer than those with private insurance, despite similar clinical profiles. This difference was mainly explained by extended delays in diagnostic testing and specialist consultations and remained significant after adjusting for comorbidities and frailty. These findings challenge the assumption that LOS reflects only clinical complexity, pointing instead to systemic inefficiencies.

Such inefficiencies are far from benign. Prolonged hospital stays increase the risk of adverse events, including medication errors, infections, and functional decline^1,2^. Each extra day raises the likelihood of complications; one study showed patients in the highest LOS percentiles had triple the mortality risk^3^. In a US cohort, nearly 25% of admissions had at least one adverse event, many of them preventable^4^. Our public cohort's average LOS (13.2 days) mirrors findings from other Latin American systems, where logistical delays often outweigh medical needs^7–9^. The high frequency of avoidable delays we observed aligns with prior reports of systemic bottlenecks in diagnostics and consultations^10^.

Several plausible mechanisms may explain the disproportionate delays observed in SUS hospitals. First, diagnostic capacity in the public sector is often constrained by equipment shortages, limited technician availability, and centralized scheduling processes^10,11^. These issues result in delayed access to basic tests such as imaging, laboratory analyses, and biopsies, which in turn delay treatment decisions and discharge. A national survey showed that delays in test results are one of the main perceived barriers to timely care in Brazil's Unified Health System^12,13^. Second, access to in-hospital specialist consultations is more limited in the public system. Staffing shortages, rigid referral protocols, and reduced availability of specialists on weekends or after hours can all contribute to longer waits^13,14^. In contrast, private hospitals tend to offer faster interdisciplinary communication and on-demand specialist input, which facilitates timely clinical decisions and discharge planning. Third, although administrative or social delays were less prevalent in our cohort, the structure of public post-discharge support services likely plays a subtle but important role. Limited coordination with community care, home health services, and equipment delivery—particularly in lower-resource regions—can prevent safe discharge and result in prolonged inpatient stays^9^. While our data show that such delays contributed minimally in terms of absolute days, their cumulative impact across large populations may be substantial.

This study has several strengths. It was conducted prospectively across multiple tertiary hospitals using real-time data collection embedded into daily clinical workflows. The categorization of delays was performed systematically by attending physicians and audited for consistency, improving data validity. Moreover, our analytic strategy—combining descriptive, stratified, and multivariate approaches—allowed us to isolate the independent contribution of systemic delays while controlling patient-level complexity. Nonetheless, some limitations warrant mention. First, although the classification of delay types was guided by predefined criteria, it relied in part on clinical judgment, which may introduce variability. Second, the study was conducted in urban tertiary centers, and findings may not generalize to rural or smaller hospitals. Third, we did not capture qualitative information about institutional workflows or staffing models, which could offer additional insights into observed disparities. Finally, although we accounted for frailty and comorbidities using validated scales, other social or functional determinants may have influenced hospitalization trajectories but remained unmeasured.

Our findings call for a reexamination of LOS as a hospital performance indicator in Brazil's dual healthcare system. When most hospital days result from operational delays rather than clinical need, LOS becomes a measure of system dysfunction. Prior studies have shown similar patterns, especially in public hospitals with limited capacity^5,6,15,16^. These inefficiencies inflate costs, block access for other patients, and increase the risk of harm^2,13^. Reducing unnecessary hospital days requires institutional reforms: expanding diagnostic capacity, streamlining specialist access, and improving discharge planning. These strategies would enhance efficiency, safety, and equity. LOS should thus be interpreted alongside other metrics—such as mortality, readmission, and patient-reported outcomes—to more accurately reflect care quality.

## Data Availability

The datasets generated and/or analyzed during the current study are available from the corresponding author upon reasonable request.
